# Characteristics of Microsatellites Mined from Transcriptome Data and the Development of Novel Markers in *Paeonia lactiflora*

**DOI:** 10.3390/genes11020214

**Published:** 2020-02-19

**Authors:** Yingling Wan, Min Zhang, Aiying Hong, Yixuan Zhang, Yan Liu

**Affiliations:** 1 College of Landscape Architecture, Beijing Forestry University, Beijing 100083, China; wan_yingling@bjfu.edu.cn (Y.W.); 18310257817@163.com (M.Z.); zyx_cyclone@163.com (Y.Z.); 2 Management Office of Caozhou Peony Garden, Heze 274000, Shandong, China; m18811581510@163.com; 3 Beijing Key Laboratory of Ornamental Plants Germplasm Innovation & Molecular Breeding, Beijing Forestry University, Beijing 100083, China; 4 National Engineering Research Center for Floriculture, Beijing Forestry University, Beijing 100083, China; 5 Beijing Laboratory of Urban and Rural Ecological Environment, Beijing Forestry University, Beijing 100083, China

**Keywords:** herbaceous peony, molecular marker, next-generation sequencing, pedigree

## Abstract

The insufficient number of available simple sequence repeats (SSRs) inhibits genetic research on and molecular breeding of *Paeonia lactiflora*, a flowering crop with great economic value. The objective of this study was to develop SSRs for *P. lactiflora* with Illumina RNA sequencing and assess the role of SSRs in gene regulation. The results showed that dinucleotides with AG/CT repeats were the most abundant type of repeat motif in *P. lactiflora* and were preferentially distributed in untranslated regions. Significant differences in SSR size were observed among motif types and locations. A large number of unigenes containing SSRs participated in catalytic activity, metabolic processes and cellular processes, and 28.16% of all transcription factors and 21.74% of hub genes for inflorescence stem straightness were found to contain SSRs. Successful amplification was achieved with 89.05% of 960 pairs of SSR primers, 55.83% of which were polymorphic, and most of the 46 tested primers had a high level of transferability to the genus *Paeonia*. Principal component and cluster dendrogram analyses produced results consistent with known genealogical relationships. This study provides a set of SSRs with abundant information for future accession identification, marker-trait association and molecular assisted breeding in *P. lactiflora*.

## 1. Introduction

Herbaceous peony, which has many varieties with distinct flower types and colors, provides great commercial benefits in the form of cut flowers and potted plants. It has a long juvenile period before flowering, which slows the development of new cultivars with specific and stable characteristics by traditional hybridization breeding [1]. Based on appropriate DNA markers, molecular-assisted breeding can be employed to select a target genotype and detect whether hybrids have the expected trait at an early stage; thus, it improves breeding efficiency and accuracy and saves time, labor and material resources [2]. The molecular breeding of herbaceous peony is not currently well developed due to a lack of foundational research; hence a large number of highly polymorphic and stable molecular markers of herbaceous peony should be developed to further identify associations with target traits.

Microsatellites, also known as simple sequence repeats (SSRs), are widely used for plant fingerprinting, genetic diversity assessment and association analysis between target traits and quantitative trait loci (QTLs) [3,4,5] due to their abundance in the genome, high polymorphism, codominant inheritance and good reproducibility [6,7]. SSRs can be developed from DNA or complementary DNA (cDNA) reverse transcribed from RNA [8]. In four previous studies on herbaceous peony, there were fewer than 16 SSR primers in each SSR-enriched genomic library or magnetic bead enrichment dataset [9,10,11]. Previous researchers synthesized 384 pairs of SSR primers from barcoded Illumina sequencing libraries of several species from the genus *Paeonia*; the researchers utilized 12 pairs of these SSRs and nine other SSR pairs to successfully identify 93 genotypes of *Paeonia* [12]. The numbers and repeat motif types of expressed sequence tag SSRs (EST-SSRs) from two sets of transcriptome data were previously reported by bioinformatics analysis, but additional PCR experiments or further validation were not performed [13,14]. Moreover, based on the transferability of SSRs among congeners of dicotyledonous plants [15], several primers from *Paeonia* were selected and used to successfully identify cultivars of *Paeonia lactiflora* [16]. Therefore, neither the number nor the application range of SSRs in herbaceous peony was not sufficient in these previous studies.

SSRs within genes or ESTs are more likely than genic SSRs obtained from SSR-enriched libraries or random DNA sequences to be effectively linked to target traits [17]. In *Populus tomentosa*, the genic SSRs selected from candidate genes related to wood formation were successfully used in family-based linkage mapping [18]. Twenty-four SSR primers of *Pisum sativum* were successfully mapped to several existing linkage groups [19]. Similar methods were also used in raspberry, in which SSRs were associated with several developmental traits [20]. For *Paeonia rockii*, SSR markers were used to perform association mapping, and 2.68–23.97% of flowering trait variance was explained [21]. Moreover, a genetic linkage map of tree peony covering five linkage groups was constructed by 124 EST-SSR primers [22]. Thus, development SSRs from herbaceous peony transcriptome may be effective for future use. Further studies showed the genomic distribution of SSRs is nonrandom. SSRs in genes may influence gene transcription or translation and gene activity [6,23], and recent studies showed a higher abundance of SSRs in response to environmental stress [24]. The polymorphism levels and potential functions of SSRs differ among the 5′ untranslated region (5′ UTR), the 3′ UTR and coding sequences (CDs) are different; SSRs in 5′ UTR may affect transcription or translation, SSRs in CDS may inactivate or activate genes, or truncate proteins, and SSRs in 3′ UTR may cause silencing or slippage [25]. However, no such information has been reported in herbaceous peony, which is not conducive to developing desirable SSR markers.

In this study, we mined SSRs from herbaceous peony transcriptome data and analyzed the distribution and location of the SSRs and the function of unigenes containing them. Initially, a total of 960 pairs of SSR primers were developed and amplified in eight cultivars from a core collection to initially validate the polymorphism level. Then, 46 pairs of primers were used to analyze transferability among nine species in *Paeonia*. Finally, we constructed a phylogenetic tree containing seven species and 24 varieties. This study provides a number of efficient and informative SSR primers for future molecular-assisted breeding of herbaceous peony.

## 2. Materials and Methods

### 2.1. SSR Identification, Annotation from Transcriptome Data and SSR Primer Design

All the SSR sequences used in this study were obtained from transcriptome data from inflorescence stems of *P. lactiflora* ‘Da Fugui’ and ‘Chui Touhong’ at five developmental stages (i.e., stages representing every seven days from stem elongation to flowering). The transcriptome data have been deposited in the Sequence Read Archive (SRA) database as described previously (accession number: PRJNA528693) [26,27] and were assembled by Trinity 3.0. MISA-web (http://pgrc.ipk-gatersleben.de/misa/) was used to search for SSRs in the unigenes [28]. The parameters were set as follows: dinucleotide (Di-) repeats had to be repeated at least 6 times, trinucleotide (Tri-) repeats had to be repeated at least five times, tetranucleotide (Tetra-) repeats had to be repeated at least four times, pentanucleotide (Penta-) repeats had to be repeated at least four times, and hexanucleotide (Hexa-) repeats had to be repeated at least four times. Interruptions were set to 100 to merge two SSR sequences into one SSR when the distance was shorter than 100 bp. Notably, mononucleotide repeats were not analyzed in this study. To identify possible SSR functions for future use, unigenes that contained SSRs were mapped to terms in the Gene Ontology (GO) database, gene numbers were calculated for each term, and the Kyoto Encyclopedia of Genes and Genomes (KEGG) database was used for pathway analysis. TransDecoder v3.0.1 (http://transdecoder.github.io/) was used to identify candidate coding regions, dividing transcript sequences into 5′ UTR, CDS and 3′ UTR sections. Primer 3 (http://bioinfo.ut.ee/primer3) [29] was used to design primers on both sides of the microsatellite sequences, following previous product size, primer length, GC content and annealing temperature principles [8].

### 2.2. Plant Materials

To evaluate the specificity and polymorphism of primers, fresh young leaves of *P. lactiflora* ‘Qihua Lushuang’, ‘Jinxing Shanshuo’, ‘Lian Tai’, ‘Fu Shi’, ‘Da Fugui’, ‘Dongfang Shaonu’, ‘Yangfei Chuyu’ and ‘Hong Fushi’ were obtained from Caozhou Peony Garden, Heze, Shandong Province, China, in April 2019. To evaluate transferability, fresh leaves of seven species of *Paeonia* were collected from different habitats in China: i.e., *P. lactiflora* was collected from Xilin Gol, Inner Mongolia (115°13′–117°06′ E, 43°02′–44°52′ N), *Paeonia emodi* and *Paeonia sterniana* were collected from Tibet (84°35′–86°20′ E, 28°3′–29°3′ N), *Paeonia obovata* was collected from Pingquan, Hebei Province (118°21′–119°15′ E, 40°24′–40°40′ N), *Paeonia anomala* was collected from Altay city (85°31′–91°04′ E, 45°00′–49°10′ N), *Paeonia intermedia* was collected from Yumin, Xinjiang Province (82°12′–83°30′ E, 45°24′–46°3′ N), and *Paeonia veitchii* was collected from Lanzhou, Gansu Province (103°40′ E, 36°03′ N). Twenty-four cultivars of *P. lactiflora* used for phylogenetic analysis were also collected from Caozhou Peony Garden. These leaves were bagged with silica gel and transported to Beijing, ground to powder with liquid nitrogen and stored at −80 °C in the laboratory of the National Engineering Research Center for Floriculture, Beijing, China. A DNAsecure plant kit (TIANGEN Biotech, Co., Ltd., Beijing, China) was used for DNA extraction. The quality and quantity of total DNA were estimated by a NanoDrop 2000 spectrophotometer (Thermo Scientific, Waltham, MA, USA). DNA was diluted to 30 ng/μL in preparation for polymerase chain reaction (PCR).

### 2.3. SSR Primer Evaluation in Eight Cultivars

A total of 960 pairs of primers were selected for synthesis (Ruibiotech, Co., Ltd., Beijing, China). To improve the efficiency of primer fluorescence labeling, the thermocycler amplification protocol was conducted in two rounds. First, the primers synthesized for the DNA of the eight cultivars were used for amplification. The 10 μL PCR mixture consisted of 0.1 μL of 10 μmol/μL forward primer containing the M13(-21) tail at its 5′ end and reverse primer, 1 μL of 30 ng/μL DNA, 5 μL of 0.1 U/μL 2×Taq PCR MasterMix (containing 0.05 units/μL Taq DNA polymerase (recombinant), 4 mM MgCl_2_ and 0.4 mM dNTPs, Aidlab Biotechnologies Co., Ltd., Beijing, China) and 3.8 μL of ddH_2_O. After an initial denaturation step of 95 °C for 5 min, 20 cycles of 95 °C for 30 s, 60 °C for 30 s and 72 °C for 30 s, as well as extension at 72 °C for 10 min, were performed. Second, to efficiently and economically analyze the length of PCR products, fluorescently labeled (i.e., FAM, HEX, TAMRA or ROX) M13(-21) universal primers were added to the PCR mix [30]. The 10 μL PCR mixture contained 0.15 μL of 10 μmol/μL M13(-21) universal primer and reverse primer, 2 μL of the PCR product from the first round, 5 μL of 0.1 U/μL 2×Taq PCR MasterMix and 2.7 μL of ddH_2_O. In the thermocycler, amplification was performed at 95 °C for 5 min and followed by 35 cycles of 95 °C for 30 s, 52 °C for 30 s and 72 °C for 30 s, as well as extension at 72 °C for 10 min. After 3% agarose gel electrophoresis, the amplified loci of the final PCR product were detected by a 3730xl DNA Analyzer with 96 capillaries (Applied Biosystems, Foster City, CA, USA) and sized with GS500 LIZ. The amplified loci were analyzed by GeneMarker V2.2.0.

### 2.4. Phylogenetic Analysis of Seven Species of Paeonia and 24 Cultivars of P. lactiflora

As shown in Table 1, the 46 forward primers showing the most abundant polymorphic loci were resynthesized by adding a fluorescent label to the 5′ tail. The 10 μL PCR mixture consisted of 0.2 μL of 10 μmol/μL forward primer and reverse primer, 1 μL of 30 ng/μL DNA template, 5 μL of 0.1 U/μL 2×Taq PCR MasterMix and 3.6 μL of ddH_2_O. PCR was performed at 95 °C for 5 min, followed by 35 cycles of 94 °C for 30 s, annealing at an appropriate temperature (as shown in Table S3) for 30 s, and 72 °C for 30 s and a final extension at 72 °C for 7 min.

Polymorphism information content (PIC) was calculated by the Microsatellite Toolkit according to the methods of a previous study [31]. GenAlEx 6.51b2 was used for genetic analysis and principal coordinates analysis (PCoA), and parameters including the number of alleles (*Na*), tests for Hardy-Weinberg equilibrium, the number of effective alleles (*Ne*), Shannon’s information index (*I*), heterozygosity (*Ho*), heterozygosity (*He*) and PIC were calculated [32,33]. The frequency of null alleles at loci were estimated by maximum likelihood method [34]. A dendrogram of the accessions of *Paeonia* was generated according to Bruvo’s distance with 1000 bootstrap replicates by the R package poppr [35].

## 3. Results

### 3.1. Numbers and Distribution of SSRs in Transcriptome Data

**Figure 1:**
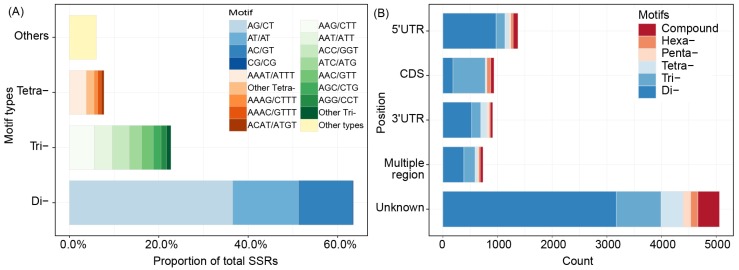
Distribution of different repeat motifs and positions of simple sequence repeats (SSRs) in unigenes. (**A**) Proportion and distribution of each type of motif in dinucleotide (Di-), trinucleotide (Tri-), tetranucleotide (Tetra-) and other (i.e., Penta-, Hexa- and compound) repeats. In the legend, ‘other Tri-’ consists of ACG/CGT (0.26%), ACT/AGT (0.26%) and CCG/CGG (0.42%), and ‘Other Tetra-’ consists of 26 types of Tetra- repeats, the most abundant of which are AATC/ATTG (0.22%) and AGGG/CCCT (0.22%). (**B**) Abundances of six motifs in different unigene positions. Two types of SSRs were located in multiple regions. One of these SSRs was located across two 5′ UTRs, a coding sequences (CDS) and a 3′ UTR; another was located in multiple CDSs in one unigene. The SSR locations differed from each other. Unknown refers to the SSRs without matching locations.

A total of 122,670 unigenes with a total length of 1.06E+08 bp were searched by MISA-web, and 10,468 SSRs (including 825 compound formations) were found. These SSRs were distributed among 8837 unigenes (7.20%), 1321 of which contained more than one SSR. As shown in Figure 1A, Di- repeats were the most abundant (63.52%) type of repeat motif, followed by Tri- repeats (22.70%), Tetra- repeats (7.73%) and all the other types of repeat motifs (6.05%). Di- repeats were of four types, namely, AG/CT (36.55%), AT/AT (14.78%), AC/GT (11.97%) and CG/CG (0.23%). The number of each type of Di- repeat (except CG/CG) exceeded the number of SSRs. TransDecoder analysis showed that these SSRs involved 4482 CDSs and 3958 unigenes. As shown in Figure 1B, the positions of many motifs were not putative, and in known positions of unigenes, different motifs exhibited distinct preferences. Di- repeats were mostly located in the 5′ UTR, followed by the 3′ UTR. In CDSs, Tri- repeats were the most abundant motif, and Tetra- repeats were mostly located in the 3′ UTR and 5′ UTR.

SSR size was analyzed as shown in Figures S1 and S2 and Table S1. For each type of repeat motif, the smallest SSRs were the most abundant, with sizes of 12 for Di- repeats, 15 for Tri- repeats, 16 for Tetra- repeats, 20 for Penta- repeats and 24 for Hexa- repeats, all of which had distinct discrete values with the increase in repeat motifs. Different types of repeat motifs exhibited significantly distinct sizes according to pairwise comparisons (Wilcoxon rank sum test, with Bonferroni adjustment). For all the SSRs, the most common size was 12 for Di- repeats (frequency of 1894), 15 for Tri- repeats (frequency of 1309) and 14 for Di- repeats (frequency of 1200). For each location (including the unknown positions) of SSRs, a large number of discrete values were observed to have high repetitions. Significant differences in the sizes of SSRs among the 5′ UTR, 3′ UTR and CDSs appeared according to pairwise comparisons (Wilcoxon rank sum test, with Bonferroni adjustment). Only the size of SSRs in the 5′ UTR differed from that in multiple regions. The size of SSRs in unknown regions was not obviously different from that in the 3′ UTR and multiple regions.

### 3.2. Annotation of the Unigenes with SSRs

**Figure 2:**
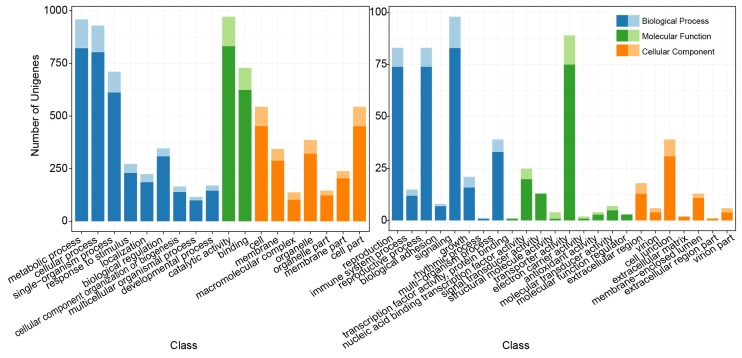
Gene Ontology (GO) analysis of unigenes containing SSRs. The lighter color of each bar represents the number of unigenes without matching coding sequences.

To understand the potential functions of the unigenes containing SSRs, we classified these unigenes using GO annotation. A total of 8837 unigenes were blasted against a protein database and annotated with GO terms. The results showed that 8522 unigenes (96.44%) were involved in three functional groups and 42 putative processes or functions, as shown in Figure 2. These unigenes participated in 17 types of biological processes, 11 types of molecular functions and 14 types of cellular components. Catalytic activity (972, 11.40% of the total blasted genes), metabolic process (959, 11.25%) and cellular process (930, 10.91%) were the three most abundant terms for putative gene functions. Fewer than ten unigenes were involved in each of the two types of biological processes, six types of molecular functions and four types of cellular components, and rhythmic process (1), transcription factor (TF) activity and protein binding (1) and extracellular region part (1) were the terms assigned the fewest unigenes containing SSRs.

**Figure 3:**
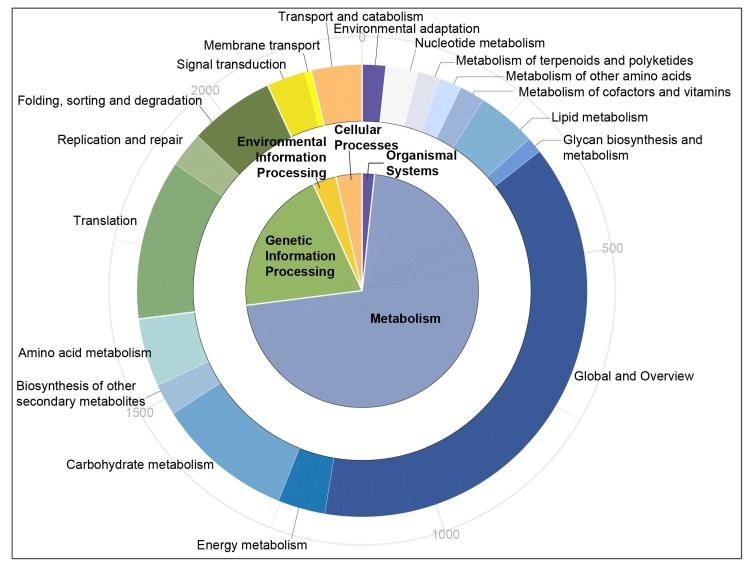
Kyoto Encyclopedia of Genes and Genomes (KEGG) pathway annotation of unigenes with embedded SSRs. The numbers outside the circle represent the cumulative number of unigenes beginning at zero and moving in a clockwise manner.

To further categorize the unigenes, KEGG annotation was used. As shown in Figure 3, unigenes with SSRs were involved in 126 pathways, which were divided into five classes and 18 subclasses. In total, unigenes participating in metabolism were most abundant. At the subclass level, the global and overview pathway (38.22%) had the largest number of unigenes, followed by the transcription pathway (11.44%), and the carbohydrate metabolism pathway (9.79%). The top five unigenes belonged to global and overview subgroups, and they were involved in metabolic pathways (15.38%), biosynthesis of secondary metabolites (8.63%), biosynthesis of antibiotics (4.11%), microbial metabolism in diverse environments (3.98%) and carbon metabolism (2.73%).

As shown in Table S2, the TFs containing SSRs accounted for 28.16% of the total TFs (1218) in the transcriptome of herbaceous peony, including 51 kinds of TF families (e.g., ERF, MYB, MYB related and ARF). Regarding the hub genes (46) for inflorescence stem straightness, as described in a previous study [27], 21.74% of the genes contained SSRs and were involved in lignin monolignol biosynthesis (*4CL1*, *CCoAOMT2*, *HST* and *CAD2*), xylan synthesis and metabolic process (*IRX-15 L*), auxin signaling transduction (*IAA26*, *IAA31* and *SAUR20*) and lateral organ boundary domain TF (*LBD15* and *LBD36*) pathways.

### 3.3. Initial Amplification of SSR Primers

Primer3 was used to design primers for the 9643 SSRs. Appropriate primers could not be designed for 2369 of these SSRs due to short or missing flanking sequences. A total of 7274 pairs of primers were designed, 3721 of which were able to identify CDSs. We further selected 960 pairs of primers considering SSR types and locations for synthesis and used them for amplification in eight distinct cultivars of *P. lactiflora*. As shown in Figure S3, 89.05% of the primers resulted in successful amplification in these cultivars, and 55.83% (i.e., 62.72% of the total with successful amplification) of the SSR marker primers had polymorphic amplification products. The total number of polymorphic loci decreased with an increasing number of alleles per locus. Appropriately 30% of the primers amplified only two or three types of products, and almost 16% of the primers amplified more than five alleles in the eight DNA templates.

### 3.4. Polymorphism in P. lactiflora

The 46 primers (listed in Table S3) with the most abundant amplified loci were used to reveal the information and transferability of the primers, as shown in Table 1.

Forty-four pairs of primers were amplified in the accessions; however, TA564 (172–201 bp) and TA566 (167–185 bp) were successful in only 26 and 25 accessions, respectively. The product size range varied among accessions. The products of T852 (209 bp) and TA144 (100 bp) had the maximum size difference among accessions, and T163 (14 bp), T237 (14 bp) and TA464 (14 bp) presented the smallest size differences among accessions. A total of 472 different alleles (Na) were amplified. The Na ranged from 6 to 16, with an average value of 10.26 ± 0.36; the Ne ranged from 2.47 to 9.96, with an average value of 5.26 ± 0.21. I varied from 1.39 to 2.41, with an average value of 1.88. The ranges of Ho and He were 0.13–0.97 (the average was 0.67) and 0.59–0.90 (the average was 0.80), respectively. SSR typing data of seven locus followed Hardy-Weinberg equilibrium, and the frequency of null alleles in other locus were 0.000–0.391 (the average was 0.11). The PIC ranged from 0.57–0.89 with an average value of 0.77.

### 3.5. Transferability of SSR Markers among Paeonia Species

**Figure 4:**
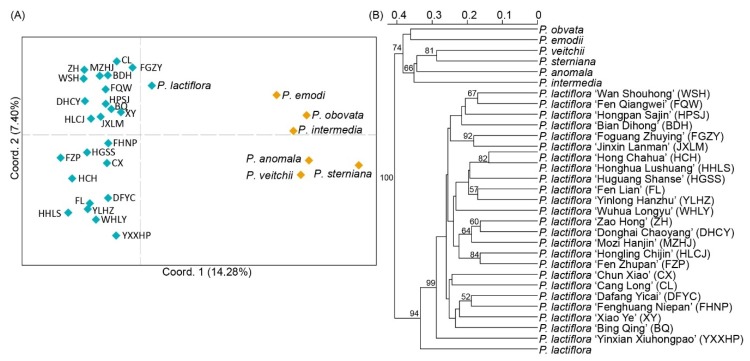
Principal coordinates analysis (PCoA) of amplified loci (**A**) and dendrogram generated by Bruvo’s distances (**B**) of 31 accessions, including seven species and 24 cultivars of *Paeonia lactiflora*. The cultivar names are abbreviated with capitalized letters in (**A**), and their full names are shown in (**B**). The UPGMA tree was produced with 1000 bootstrap replicates, and the node values greater than 50 are shown in the tree.

PCoA of 31 accessions was conducted according to the amplified alleles, as shown in Figure 4A. Eigen values by axis and sample eigen vectors are shown in Table S4. A total of 30 dimensions were extracted, and dimension 1 (10.7%) and dimension 2 (7.1%) represented 17.8% of the total information. The points representing the 24 cultivars of *P. lactiflora* were close to each other and separated from points of the other seven species. Of these species, *P. lactiflora* was nearest to the 24 cultivars in the PCoA plot, and *P. intermedia* was significantly separated from all the accessions. A cluster dendrogram was drawn according to Bruvo’s distances calculated by the amplified alleles, as shown in Figure 4B. All the accessions were divided into two groups, and the species *P. lactiflora* and its cultivars were tightly clustered and separated from the other *Paeonia* species, which was consistent with the results of PCoA. Furthermore, at a height of 0.85, *P. obovata* and *P. emodi* were further clustered and separate from the other four species. Among the cultivars of *P. lactiflora*, ‘Yinxian Xiuhongpao’ had the maximum distance from the other cultivars and was separated at a height of 0.65.

## 4. Discussion

Next-generation sequencing makes it possible to develop microsatellites efficiently and inexpensively [8]. In this study, we identified a total of 10,468 SSRs, covering 7.20% of the transcripts assembled using our transcriptome data. This method was significantly more convenient and effective than the use of SSR-enriched genomic libraries or magnetic bead enrichment, which were the primary methods used in previous herbaceous peony studies [9,10,11,36,37]. The coverage of SSRs in ESTs reported in the present study was higher than that reported in five cereals (average of 3.2%) [38] and similar (6.6%) to that generated by Trinity for *P. lactiflora* ‘Hang Baishao’ [13]. The distribution of motif types varies among plants. The most frequent motif type for *Parrotia subaequalis* was Di- [39], while the most frequent motif type for *Lychnis kiusiana* and *Dendrocalamus hamiltonii* was Tri- [40,41]. In this study, Di- repeats were the most abundant motif (mononucleotides were not considered) in *P. lactiflora*, and AG/CT accounted for 36.55% of all SSRs, followed by AT/AT (14.78%) and AC/GT (11.97%). The observation that AG/CT was the most frequent repeat was consistent with the finding of a previous study in the genus *Paeonia* regardless of assemble methods [13], while the proportions of TC/GA and AC/GT repeats significantly differed [14,42]. Differences in the transcriptomic SSR motifs can explain the relatively low transferability of SSR primers (approximately 26%) from the genus *Paeonia* to *P. lactiflora* [16]. 

SSR size varied significantly among unigene locations and motif types in this study. Furthermore, our results suggested distinct preferences in the distribution of motifs among different gene parts; in annotated positions, a large number of Di- repeat motifs were distributed in the 5′ UTR and 3′ UTR, and most of the Tri- repeat motifs were distributed in CDSs. Polymorphism level is affected by location; notably, a large proportion of SSRs in the 3′ UTR were polymorphic in *Hordeum vulgare* [43]. Further studies in grape showed that the most polymorphic SSR position differed at three levels, that is, among cultivars, among cultivars and species, and among species and genera [25]. As shown in Figure S3, in the initial screening of 960 SSR primers in eight cultivars, we found that at the cultivar level, SSRs from the 5′ UTR (64.05%) were the most polymorphic, followed by those from the 3′ UTR (60.61%). This result suggested that the polymorphism level of SSR locations was related to species.

SSRs from the transcribed sequence may be directly related to phenotypic variation and thus related to functional trait. SSR alleles associated with biotic or abiotic stress, such as heat, cold, salt and resistant to multiple diseases have been reported [44,45,46,47]. SSRs from specific organ are likely to associate with corresponding morphological traits; SSRs obtained from flower bud transcriptome in *P. rockii* have been demonstrated significantly associating with flower colors and shapes [21]. In this study, SSRs were investigated from transcriptome that was obtained from two cultivars with distinct straightness of inflorescence stem, and a large quantity of unigenes with SSRs were annotated with the catalytic activity, metabolic process and cellular process terms. Furthermore, 28.16% of all TFs and 21.74% of the hub genes for inflorescence stem straightness contained SSRs. We speculate that these SSRs likely associated with straightness characteristics of the herbaceous peony inflorescence stem, while association analysis and QTL mapping are needed in further study.

In our experiment, 89.05% of 960 pairs of primers were validated by PCR, and 55.83% of the primers were polymorphic, which was approximately the percentage (59.90%) previously reported in peony [10], higher than the percentage (36.67%) in *Amentotaxus argotaenia* [48], and lower than the percentage for SSR markers (77.2%) generated from the soybean genome [49]. These amplification differences may be due to the number of individuals used for amplification or locus mutations (e.g., insertions, deletions and translocations) among species or cultivars [50]. To identify the reason, more individuals should be subjected to PCR amplification, and cloning experiments and sequencing should be carried out.

The mean Na in this study was 10.26, and Ho and He were 0.67 and 0.80, respectively, which were higher than the Ho and He reported in previous studies on tree peony and herbaceous peony [9,10,51]. The mean PIC value was 0.77, showing a high level of high informativeness [52]; compared to mean PIC value (0.4149–0.678) revealed by previous SSR development [9,10,36,37], our results were significantly higher, suggesting our contribution for future effective genetic analysis or QTL mapping of *Paeonia* with fewer SSRs. Previous studies suggested that the presence of null alleles is common, and it has influence on evaluation of genetic diversity of population, even causes misunderstanding in parentage analysis [53,54]. Literature showed that the frequencies of null alleles were almost fewer than 0.40 and most of them were fewer than 0.20 [55]. Our results showed the frequencies of null alleles of 22 SSRs were less than 0.08 (or no presence), and only that of four SSRs were between 0.20 and 0.40. The future use of these SSRs should carefully consider the influence of null alleles according to research objective and choose the appropriate SSRs.

EST-SSRs developed for one species can be transferred to related species, with transferability varying depending on the plant and SSR source. In *Magnolia wufengensis* and *Elymus sibiricus*, the transferability of EST-SSRs to related species was 50–68.1% and 49.1%, respectively. In SSRs from candidate genes of *Oryza sativa*, transferability ranged from 70.37% to 77.78% according to different complexes [56]. In this study, 52.17% of the 46 pairs of SSR primers selected from the initial screening could be completely transferred to seven species of the genus *Paeonia*, 39.13% of the pairs had high transferability (six or five of seven accessions were successfully amplified), and 4.35% of the pairs had moderate transferability and could be amplified in four of seven species.

The diversity of dimensions extracted from PCoA and the low explanatory power of one dimension suggested that the genetic background of the *Paeonia* accessions involved in this study varied greatly. Combining the PCoA plot and the dendrogram of 31 accessions, the genetic relationships between these accessions were almost consistent with their recognized morphological classification [57,58]. These SSR markers can be used in genetic variance analysis and to initially evaluate the value of breeding parents selected according to genetic distance in the genus *Paeonia*. 

## 5. Conclusions

In this study, a large quantity of informative SSRs were conveniently identified from transcriptome data of *P. lactiflora*, and the distribution and location of motifs were defined. SSR containing genes associated with TFs and inflorescence stem straightness were identified, providing a foundation for future marker-trait association research. To the best of our knowledge, this is the first study to comprehensively reveal the characteristics and functional annotations of EST-SSRs in *P. lactiflora*. In future studies, more herbaceous peony accessions should be tested to further evaluate the polymorphism of markers, and more functional markers potentially associated with traits should be developed to advance the molecular breeding of *P. lactiflora*.

## Figures and Tables

**Table 1:** Size range of amplification products, sample size (N), the frequency of null allele at locus (Null allele), number of different alleles (Na), number of effective alleles (Ne), Shannon’s information index (I), observed heterozygosity (Ho), expected heterozygosity (He), fixation index (F) and polymorphism information content (PIC)./means we did not calculated the frequency of null allele at the locus because there was no significant difference in the observed and expected value in this locus and it followed Hardy-Weinberg equilibrium (Chi-Square tests, *p* < 0.05).

Locus	Repeat Motif	Size Range (bp)	N	*Na*	*Ne*	*I*	*Ho*	*He*	*Null allele*	F	PIC
T125	(TC)9	128–170	31	15	7.84	2.29	0.77	0.87	0.073	0.11	0.86
T163	(TA)8	146–160	31	8	3.96	1.63	0.65	0.75	0.085	0.14	0.71
T237	(CT)10	95–109	31	8	4.59	1.76	0.68	0.78	0.095	0.13	0.75
T241	(GA)15	127–157	31	12	6.77	2.11	0.81	0.85	/	0.05	0.84
C160	(AT)9ctcctt(CTC)5	211–227	29	8	4.58	1.79	0.66	0.78	0.117	0.16	0.76
TA564	(TA)8	172–201	26	9	5.43	1.90	0.77	0.82	0.058	0.06	0.79
T317	(CT)10	157–183	30	9	5.94	1.92	0.83	0.83	/	0.00	0.81
S024	(AAT)16	161–201	31	10	5.60	1.94	0.77	0.82	0.042	0.06	0.80
T040	(GA)7	228–246	31	7	5.02	1.73	0.68	0.80	0.106	0.15	0.77
T179	(AT)7	235–269	31	10	4.93	1.83	0.71	0.80	0.070	0.11	0.77
T192	(CT)8	212–230	31	8	4.50	1.75	0.68	0.78	0.093	0.13	0.75
T300	(AT)10	218–238	31	9	4.45	1.80	0.48	0.78	0.171	0.38	0.75
TA673	(CA)18	190–288	31	15	6.74	2.20	0.81	0.85	0.058	0.05	0.84
T210	(GA)10	253–285	31	12	4.44	1.86	0.81	0.77	0.044	−0.04	0.75
TA038	(CT)9	259–299	31	13	6.74	2.18	0.13	0.85	0.391	0.85	0.84
S033	(TAT)7	137–181	31	10	2.47	1.39	0.42	0.59	0.123	0.29	0.57
T304	(GA)10	147–163	30	9	4.75	1.77	0.63	0.79	0.144	0.20	0.76
T863	(AG)10	140–160	31	10	6.26	2.03	0.74	0.84	0.070	0.12	0.82
TA028	(AC)6	101–123	31	8	3.88	1.69	0.48	0.74	0.177	0.35	0.72
T239	(CT)9	185–221	31	11	2.98	1.59	0.58	0.66	0.069	0.13	0.64
TA144	(CT)6	186–286	31	9	4.00	1.64	0.68	0.75	/	0.10	0.71
T852	(AT)8	158–367	31	13	5.95	2.06	0.35	0.83	0.268	0.57	0.81
F106	(TATG)5	200–290	31	13	5.02	2.03	0.55	0.80	0.145	0.32	0.78
TA082	(AG)6	242–280	30	13	6.00	2.03	0.73	0.83	0.084	0.12	0.81
TA079	(AT)8	232–264	29	8	2.93	1.45	0.52	0.66	0.161	0.21	0.62
T356	(AG)9	247–269	30	9	4.76	1.81	0.33	0.79	0.296	0.58	0.76
TA133	(AT)8	258–286	28	9	4.28	1.72	0.75	0.77	0.095	0.02	0.73
SA010	(AAT)5	264–294	31	9	4.98	1.85	0.71	0.80	0.079	0.11	0.77
W75	(CTCAC)5	257–293	31	8	4.65	1.70	0.65	0.79	0.092	0.18	0.75
T205	(TC)9	275–301	31	13	6.94	2.18	0.71	0.86	/	0.17	0.84
S853	(TTG)5	158–186	29	8	4.67	1.71	0.69	0.79	/	0.12	0.75
T859	(AG)19	166–200	29	11	7.61	2.15	0.90	0.87	0.000	-0.03	0.85
TA566	(GA)6	167–185	25	7	4.83	1.71	0.80	0.79	/	−0.01	0.76
TA695	(AC)15	164–180	30	8	3.00	1.49	0.57	0.67	0.119	0.15	0.64
S237	(CTG)8	193–219	31	13	9.96	2.41	0.74	0.90	0.081	0.18	0.89
TA464	(TC)8	200–214	30	8	5.56	1.85	0.73	0.82	0.062	0.11	0.80
T160	(CT)10	205–227	30	10	5.47	1.92	0.70	0.82	/	0.14	0.80
TA134	(GA)16	142–224	29	13	5.43	2.09	0.93	0.82	0.019	−0.14	0.80
SA061	(GAA)7	257–277	29	6	3.43	1.46	0.38	0.71	0.281	0.46	0.67
TA074	(CT)8	260–306	30	13	5.79	2.03	0.97	0.83	0.000	−0.17	0.81
TA704	(TC)17	237–277	30	7	5.19	1.73	0.70	0.81	0.111	0.13	0.78
TA610	(TC)8	257–291	28	11	6.25	2.05	0.68	0.84	0.117	0.19	0.82
S025	(ATT)7	268–350	29	13	5.14	2.02	0.55	0.81	0.200	0.32	0.79
TA022	(TC)6	276–318	30	16	6.87	2.23	0.90	0.85	0.000	−0.05	0.84
TA086	(AG)10	393–419	31	10	5.62	1.94	0.74	0.82	0.077	0.10	0.80
TA700	(AG)9	278–312	29	13	5.76	2.05	0.62	0.83	0.155	0.25	0.81
Mean			30.07	10.26	5.26	1.88	0.67	0.80	0.11	0.16	0.77
SE			0.20	0.36	0.21	0.03	0.02	0.01	0.01	0.03	0.01

## Supplementary Materials

The following are available online at https://www.mdpi.com/2073-4425/11/2/214/s1, Figure S1: Size distribution of SSRs with different motifs and different unigene positions. (A) SSR size of different motifs. Considering the data coverage and to improve the readability of the graph, the figure shows 99% of the data. The distribution of all data is shown in Figure S2. Size differences between motifs were analyzed with pairwise comparisons using the Wilcoxon rank sum test (after Bonferroni correction), and all the outputs were less than 2.2 × 10^−16^. (B) Sizes of SSRs in different unigene positions. The figure shows 98% of the data. The distribution of all data is shown in Table S1. Size differences between positions were compared using the same method as in (A), and the figure shows combinations with p-values less than 0.05, Figure S2: Size distribution of each type of repeat motif in all data, Figure S3: Polymorphism rate of primers designed for different positions of SSRs in unigenes, Table S1: Numbers of SSRs with distinct sizes in different regions, Table S2: Count of SSRs in TFs, Table S3: Sequence, position, repeat motif, annotation, annealing temperature and amplified product size of 46 SSR primers, Table S4: Eigen values by axis and sample eigen vectors.
