# Supplementary figures and images for: Characteristics of Microsatellites Mined from Transcriptome Data and the Development of Novel Markers in Paeonia lactiflora
Source: Genes (Basel). 2020 Feb 19;11(2):214. doi: 10.3390/genes11020214 (PMC7073652; doi:10.3390/genes11020214)

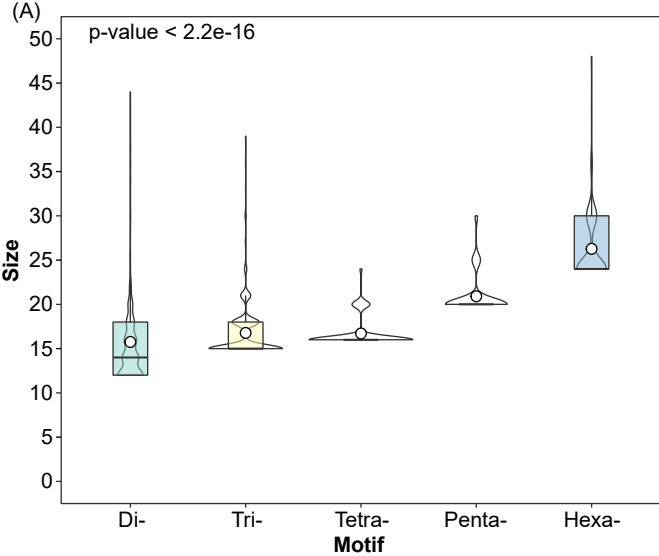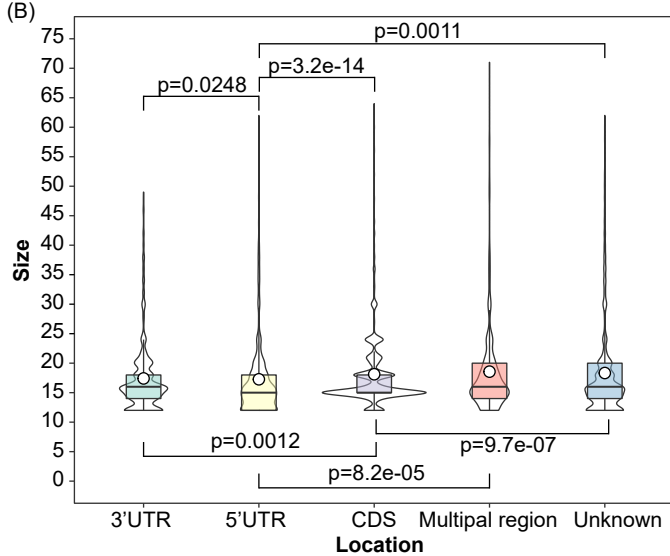

Supplement: Supplementary file 1 [file genes-11-00214-s001.zip › Supplementary/FigureS1.pdf]

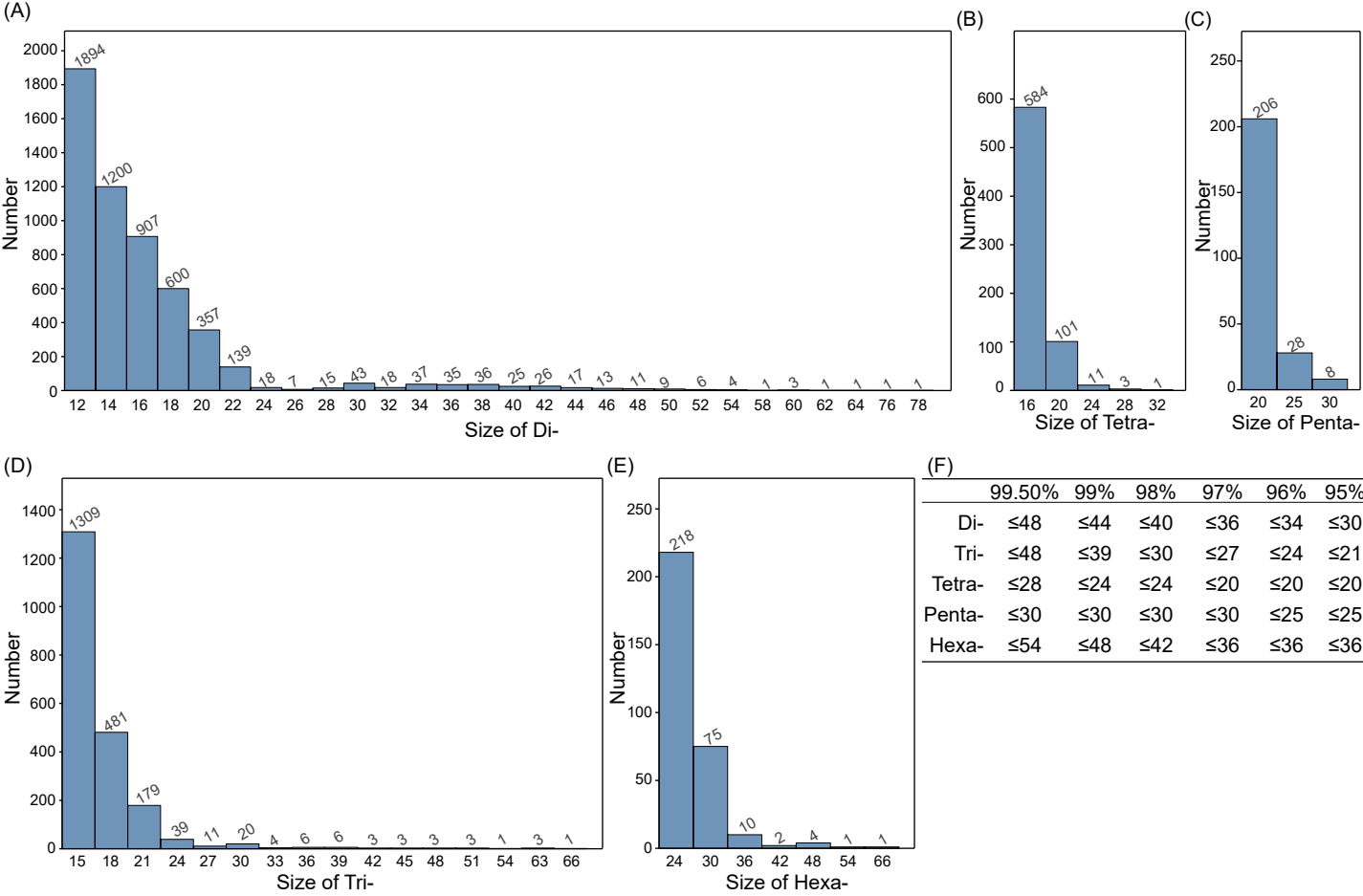

Supplement: Supplementary file 1 [file genes-11-00214-s001.zip › Supplementary/FigureS2.pdf]

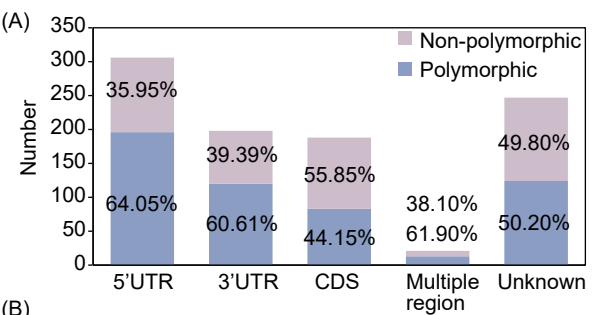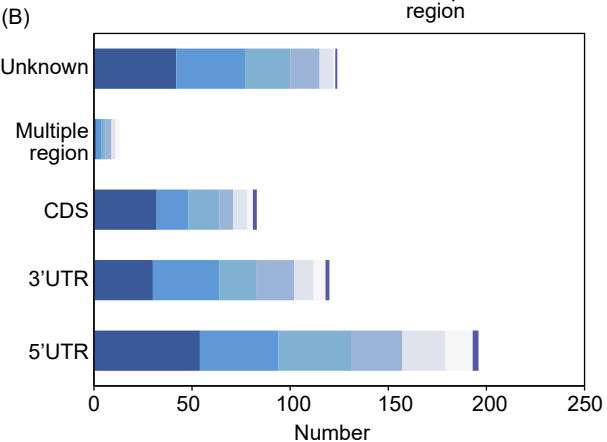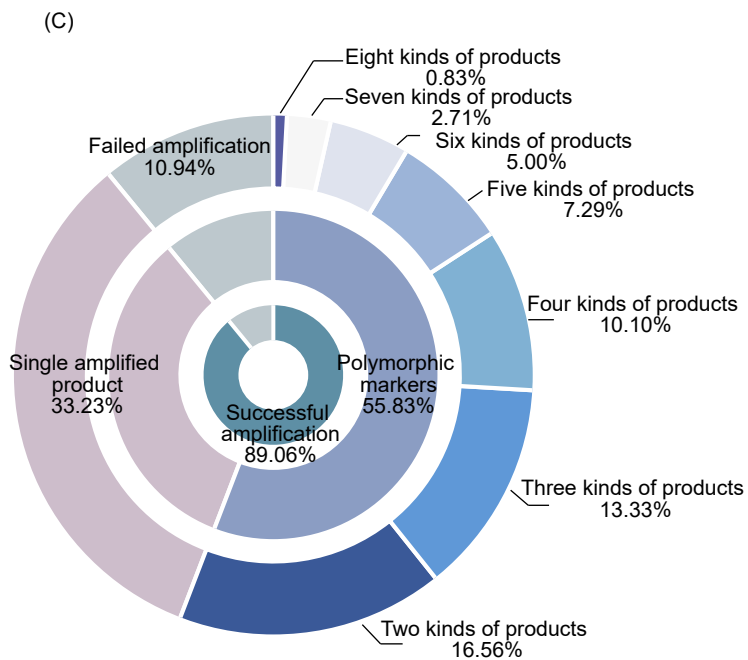

Supplement: Supplementary file 1 [file genes-11-00214-s001.zip › Supplementary/FigureS3.pdf]
